# The Helicobacter pylori UvrC Nuclease Is Essential for Chromosomal Microimports after Natural Transformation

**DOI:** 10.1128/mbio.01811-22

**Published:** 2022-07-25

**Authors:** Florent Ailloud, Iratxe Estibariz, Gudrun Pfaffinger, Sebastian Suerbaum

**Affiliations:** a Medical Microbiology and Hospital Epidemiology, Max von Pettenkofer Institute, Faculty of Medicine, LMU Munich, Munich, Germany; b German Center for Infection Research (DZIF), Partner Site Munich, Munich, Germany; Rutgers, The State University of New Jersey

**Keywords:** DNA recombination, *Helicobacter pylori*, genome analysis, nucleotide excision repair

## Abstract

Helicobacter pylori is a Gram-negative bacterial carcinogenic pathogen that infects the stomachs of half of the human population. It is a natural mutator due to a deficient DNA mismatch repair pathway and is naturally competent for transformation. As a result, it is one of the most genetically diverse human bacterial pathogens. The length of chromosomal imports in H. pylori follows an unusual bimodal distribution consisting of macroimports with a mean length of 1,645 bp and microimports with a mean length of 28 bp. The mechanisms responsible for this import pattern were unknown. Here, we used a high-throughput whole-genome transformation assay to elucidate the role of nucleotide excision repair pathway (NER) components on import length distribution. The data show that the integration of microimports depended on the activity of the UvrC endonuclease, while none of the other components of the NER pathway was required. Using H. pylori site-directed mutants, we showed that the widely conserved UvrC nuclease active sites, while essential for protection from UV light, one of the canonical NER functions, are not required for generation of microimports. A quantitative analysis of recombination patterns based on over 1,000 imports from over 200 sequenced recombinant genomes showed that microimports occur frequently within clusters of multiple imports, strongly suggesting they derive from a single strand invasion event. We propose a hypothetical model of homologous recombination in H. pylori, involving a novel function of UvrC, that reconciles the available experimental data about recombination patterns in H. pylori.

## INTRODUCTION

Helicobacter pylori is a Gram-negative bacterial pathogen colonizing the stomach mucosa of approximately one half of the human population (1, 2). The infection is typically acquired during childhood by person-to-person transmission and persists for the lifetime of its host unless treated. H. pylori lacks the canonical DNA mismatch repair (MMR) pathway and is naturally competent for transformation (3, 4). As a result of these and other species-specific features of its genetic machinery, H. pylori is one of the most genetically diverse human pathogens and therefore a valuable model to understand within-host adaptation (5, 6). The human stomach is the sole reservoir of H. pylori, and mixed infections with multiple H. pylori strains likely represent the only opportunity for interstrain recombination. Exogenous DNA is taken up from the environment by the ComB apparatus, which is related to type IV secretion systems (7, 8). It is then translocated into the cytoplasm as single-stranded DNA (ssDNA) through the ComEC channel (9, 10). The incoming DNA is subsequently integrated into the chromosome of the recipient by homologous recombination (HR).

The genetic diversity introduced by HR in naturally competent bacteria is shaped by both the recombination rate and the tract length of imported DNA fragments. In particular, the recombination tract length (import length) differs greatly across species. Median import lengths of 10,000 bp in Bacillus subtilis (11) and 7,000 bp in Haemophilus influenzae (12) have been observed experimentally *in vitro*. In clinical isolates, import lengths of 5,000 bp were detected in *Neisseria* spp. (13). Understanding how import length is determined has important implications for understanding the evolution and maintenance of natural competence. Indeed, HR can offer selective advantages, either by bringing together beneficial mutations, such as alleles related to antibiotic resistance (14, 15), or by purging deleterious alleles (16, 17). In the latter case, HR plays an important role in preventing genome deterioration through Muller’s ratchet, in particular for organisms with small within-host effective population sizes such as H. pylori (18, 19). On the other hand, HR can also carry a high genetic fitness cost since extracellular DNA in the environment might derive from dead cells affected by deleterious mutations (20). Consistent with this, the import length is a crucial factor in modulating the genetic load of the transforming DNA pool and directly affects the impact of HR on the fitness of bacterial populations.

In H. pylori, HR might contribute to the balance required for long-term persistence in the human gastric niche, and significantly shorter import lengths were estimated from either patient isolates (21–23) or *in vitro* transformation assays (24, 25). Import patterns in H. pylori were shown to follow a bimodal distribution consisting of a 1:9 mixture of relatively rare microimports with a mean length of 28 bp and more frequent macroimports with a mean length of 1,645 bp (26). This observation was achieved using whole-genome sequencing to detect recombination from *in vitro* transformants along the entire chromosome of H. pylori. For most naturally competent bacteria, recombination has not been characterized on a whole-genome scale, and bimodality of the import length distribution has so far only been observed by genome sequencing in H. influenzae (12) and Streptococcus pneumoniae (27). In H. influenzae, microimports have not been characterized further. In S. pneumoniae, the MMR pathway typically limits recombination, leading to microimports, but is thought to become saturated when the incoming DNA is too divergent from the recipient, which then leads to macroimports (28). A wide range of import lengths might help maintain the equilibrium between the benefit and disadvantages of HR under changing environmental conditions by controlling the genetic load of the pool of available transforming DNA. In H. pylori, the MMR system is absent, and therefore the current study was designed to elucidate the mechanisms responsible for bimodality in this organism.

While canonical pathways of HR are fairly well described in several prokaryotes (29), many steps have not been experimentally demonstrated in H. pylori. The current model in H. pylori states that internalized ssDNA is first coated by the RecA and DprA proteins, forming the presynaptic complex (30, 31). The resulting DprA-RecA-ssDNA filament then searches for sequence homology along the double-stranded DNA (dsDNA) and initiates strand invasion, leading to the formation of a three-stranded displacement loop (D-loop). During postsynapsis, the branches of the D-loop are extended via branch migration mediated by the helicase RecG and subsequently resolved by the endonuclease RuvC (32). Natural transformation in H. pylori appears to be intertwined with DNA repair since several enzymes of the base excision repair (BER), nucleotide excision repair (NER), and recombinational repair pathways influence either recombination frequency (33–38) and/or the import length (24, 39, 40). So far, none of the proteins related to the postsynaptic steps appears to be strictly required for natural transformation, suggesting that several redundancies most likely exist in the HR process.

Using a high-throughput whole-genome transformation assay, we characterized the distribution of import lengths among distinct H. pylori genetic backgrounds and analyzed the role of the NER pathway in HR. In particular, we demonstrate that integration of microimports is dependent on the UvrC endonuclease. We go on to show that the nuclease active sites and the DNA-binding domains of UvrC were not involved in this mechanism. The C-terminal DNA-binding region of UvrC has an atypical structure in H. pylori, as well as other *Helicobacter* spp., compared to model organisms such as Escherichia coli or Thermotoga maritima, and it does not contribute to UV tolerance, suggesting functional divergence. Furthermore, we quantitatively analyzed the clustering of imports on the chromosome and show that microimports integrated via UvrC occur frequently within clusters of imports. Finally, we propose an HR model reconciling our observations about recombination patterns in H. pylori.

## RESULTS

### A bimodal distribution of import lengths is conserved in H. pylori.

**FIG 1:**
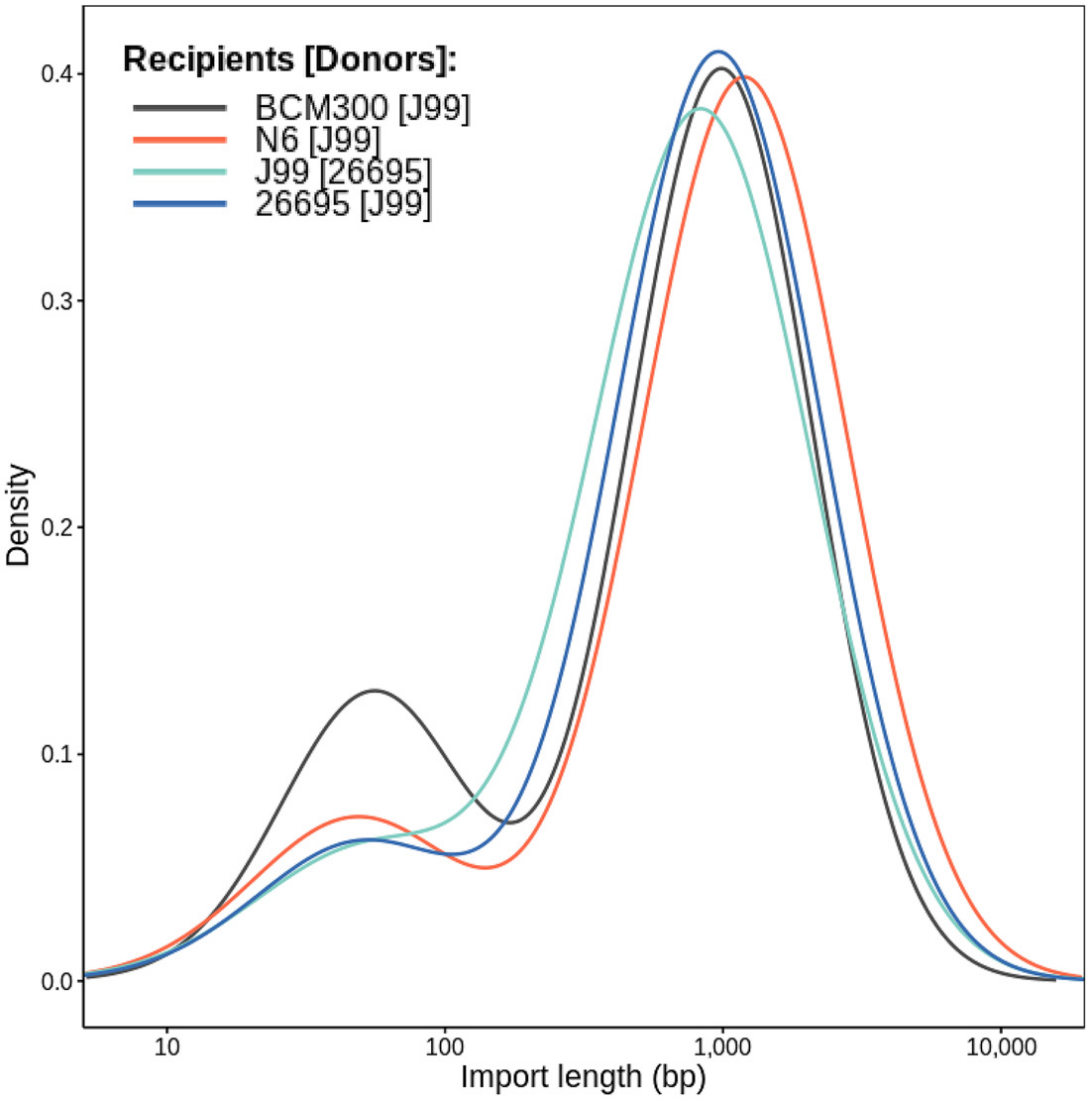
Distribution of import lengths in four recipient-donor pairs of wild-type H. pylori strains. The probability density of import lengths was estimated using a normal-mixture model. The import length is represented on the *x* axis using a logarithmic scale. The *y* axis represents the density of probability for any given import length.

A bimodal distribution of import lengths after natural transformation of H. pylori with homeologous genomic DNA from a different H. pylori strain was initially observed with the hpEurope strain 26695 as a recipient and the hpAfrica1 strain J99 as a donor, in experiments involving either a single round of transformation (short-term transformation [STT] protocol) or multiple transformation cycles (TC protocol) (26). The current study was conceived to identify mechanisms involved in generating this bimodal distribution. Because the throughputs of STT and TC assays are too limited to efficiently characterize multiple H. pylori mutants, we modified the STT protocol with the aim to improve both its throughput and sensitivity. Transformation was performed on blood agar plates instead of liquid media, shortening the culture time by 48 h. The quantity of donor genomic DNA (gDNA) was scaled down by a factor of 50, and a 1:10 mixture of marked and unmarked gDNA was added to the recipient strain instead of marked gDNA only. As determined previously, imports transferred in the vicinity of the antibiotic resistance marker were noticeably longer than those randomly distributed in the genome (26). Therefore, such imports were systematically excluded from the analysis to eliminate this bias and increase the sensitivity of the assay. Performed with the same recipient-donor pair of H. pylori strains, the novel plate STT (pSTT) protocol generated a bimodal distribution similar to the original STT method (Fig. 1). This distribution was composed of a peak of microimports with an average length of 51 bp and a peak of macroimports with an average length of 962 bp. Microimports represented 13% of all observed imports.

Considering the heterogeneity of H. pylori, the distribution of import lengths may be variable between strains. In order to evaluate the stability of the import lengths, we tested additional recipient-donor combinations, mixing distinct genetic backgrounds: J99-26695, N6 (hpEurope)-J99, and BCM300 (hpAfrica1)-J99. The bimodal distribution was observed in all tested pairs (LRT, *P* < 0.05) with some fluctuation. The proportion of microimports ranged from 13% to 24%, while the mean lengths of micro- and macroimports ranged from 48 to 56 bp and 833 to 1,200 bp, respectively (Fig. 1). The experimental variation inherent to transformation assays is partly responsible for the fluctuation in those parameters, albeit the sequence divergence of each recipient-donor pair may also have an effect. Notably, the BCM300-J99 pair, which consists of two hpAfrica1 strains, was the least divergent and displayed the highest proportion of microimports. Altogether, these results indicate that import patterns in H. pylori are most likely conserved across the species.

### The integration of microimports requires the presence of the nuclease UvrC, but not other components of the NER pathway.

Each major DNA repair mechanism described in H. pylori has been shown to have an influence on natural transformation. In particular, different enzymes of the NER pathway have been associated with a hyperrecombinational phenotype and/or the integration of longer imports (40). NER is found in both eukaryotes and prokaryotes (41, 42) and is the only DNA repair mechanism omnipresent in bacterial pathogens (43, 44). This pathway is responsible for the repair of bulky DNA lesions, such as those caused by UV light, based on the distortion of the DNA double helix (45). In E. coli and other prokaryotes, the NER system consists of four proteins, UvrA to UvrD. Damage recognition is carried out by a protein complex made up of UvrA and UvrB subunits. After binding the lesion, UvrA dissociates from the complex and is replaced by UvrC, which catalyzes incisions on both sides of the lesion. The damaged segment is then excised by the helicase UvrD and the resulting gap filled by the DNA polymerase I and DNA ligase. In H. pylori, the NER pathway is involved in DNA repair of damages induced by low pH (46) and UV light (40) but has not been exhaustively characterized.

**FIG 2:**
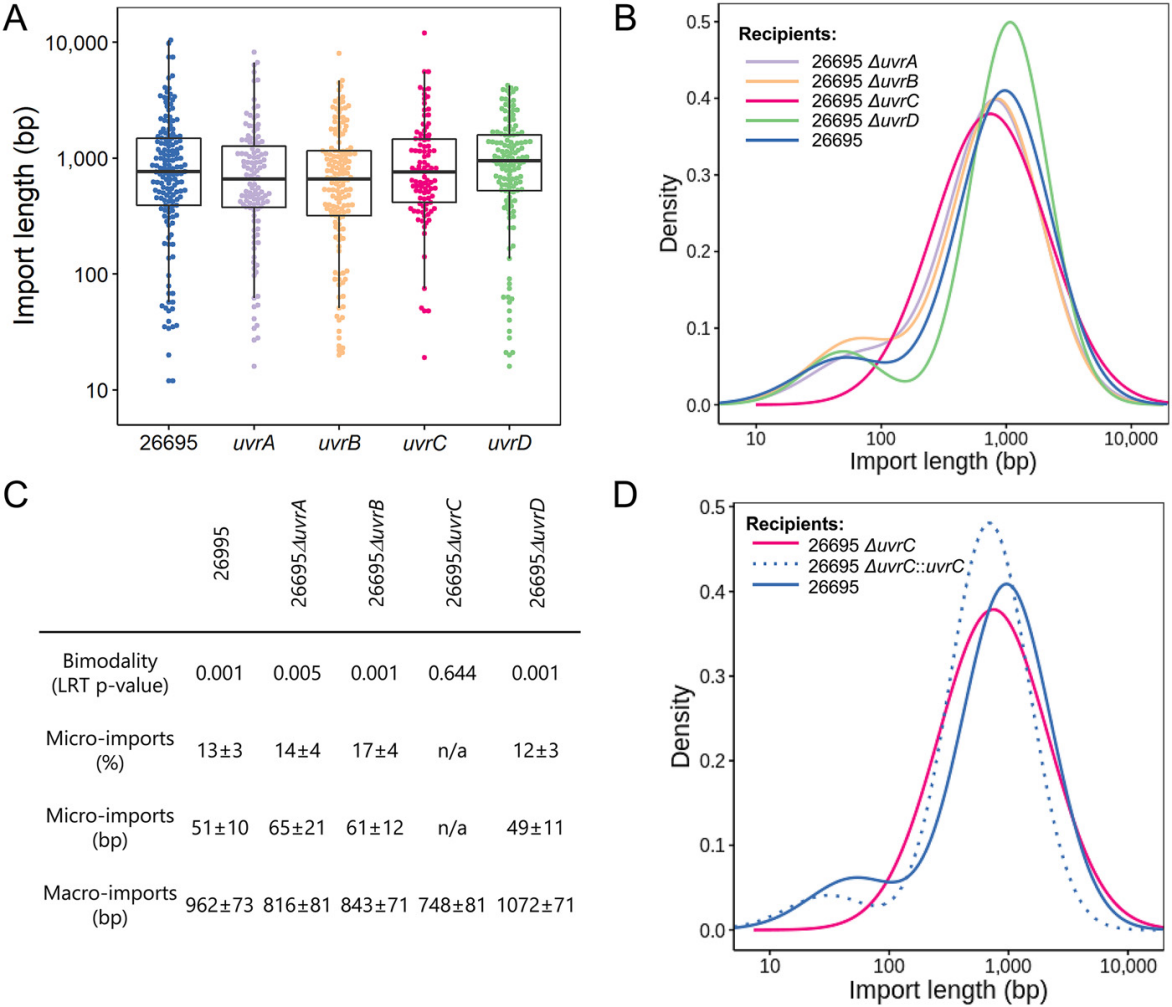
Influence of components of the NER pathway on the distribution of import lengths in H. pylori. (A) Distribution of import lengths in the individual NER knockout mutants of the NER pathway. Each point represents an import. Whiskers indicate the minimum and maximal values. Boxes show the interquartile range. The central line represents the median. (B) Density estimation of import lengths in the individual NER knockout mutants of the NER pathway; (C) Parameters of the distribution of import lengths in individual knockout mutants of the NER pathway. Bimodality represents the *P* value from a likelihood ratio test obtained for a model with two mixture components. Mean ± standard errors from bootstrap resampling are indicated. (D) Density estimation of import lengths in the complemented *uvrC* knockout mutant.

To investigate a potential contribution of this pathway to the bimodal import pattern, individual knockout mutants of *uvrA*, *uvrB*, *uvrC*, and *uvrD* were characterized in strain 26695 by the pSTT method. The import pattern was unaffected in the *uvrA*, *uvrB*, and *uvrD* mutants. In contrast, the distribution of import lengths in the *uvrC* knockout was unimodal (Fig. 2A and B). The imports observed in the UvrC mutant had a mean length of 748 bp with minimum and maximum lengths of 19 and 12,024 bp, respectively (Fig. 2C). The single peak in this unimodal distribution was comparable to the peak of macroimports observed in the wild type, whereas the peak of microimports was absent (Fig. 2B). To confirm the effect of UvrC, a complemented strain was created for the 26695 mutant, and knockouts were generated in two additional strains, J99 and N6. The bimodal distribution was restored in the complemented strain, although with a smaller proportion of microimports (8%) compared to the wild type (13%) (Fig. 2D). In strains J99 and N6, the deletion of *uvrC* resulted in a similar shift of the import lengths’ distribution than obtained in the strain 26695 (see Fig. S1 in the supplemental material).

10.1128/mbio.01811-22.2 FIG S1 Distribution of import lengths in *uvrC* knockout mutants in three H. pylori strains. (A) Strain J99; (B) strain N6. The probability density of import lengths was estimated using a normal-mixture model. The import length is represented on the *x* axis using a logarithmic scale. The *y* axis represents the density of probability for any given import length. Download FIG S1, PDF file, 0.05 MB. Copyright © 2022 Ailloud et al. 2022 Ailloud et al. https://creativecommons.org/licenses/by/4.0/ This content is distributed under the terms of the Creative Commons Attribution 4.0 International license.

In the absence of UvrC, the single peak of macroimports observed in the mutants is likely the result of the canonical pathway of HR, previously described in H. pylori (6, 32). Because the boundaries of imports appear to be determined by a stochastic process in H. pylori (26), fragments unusually shorter or longer than the average macroimport length can be integrated randomly at low frequency. In particular, the shortest imports observed at low frequency in the *uvrC* mutants have a similar length to the imports observed in the peak of microimports from the wild-type strains. These observations suggest that UvrC is involved in a mechanism increasing the probability of integration for short fragments, resulting in the peak of microimports. Compared to the import length distribution formed by the canonical pathway, this increase promoted by UvrC might have been relevant for the evolution of H. pylori.

### UvrC catalytic sites are not required for the integration of microimports.

The endonuclease UvrC has been characterized in several different organisms, such as E. coli (47–51) or T. maritima (52, 53). Within the NER pathway as described in these species, UvrC carries out a dual incision 3′ and 5′ of a DNA lesion, using two independent endonuclease domains. The amino-terminal catalytic site is part of the GIY-YIG endonuclease superfamily and responsible for the 3′ incision, while the 5′ incision is performed by the carboxy-terminal site that shares homology with the RNase H family. Both incisions are partially dependent of a DNA-binding domain connected to the carboxy-terminal region by a flexible linker and harboring a pair of helix-hairpin-helix (HhH) motifs (51). Additionally, a UvrB-interacting domain is located between the two catalytic sites and is required for the 3′ incision (48, 54). For H. pylori UvrC, similar functional analyses have not been performed.

**FIG 3:**
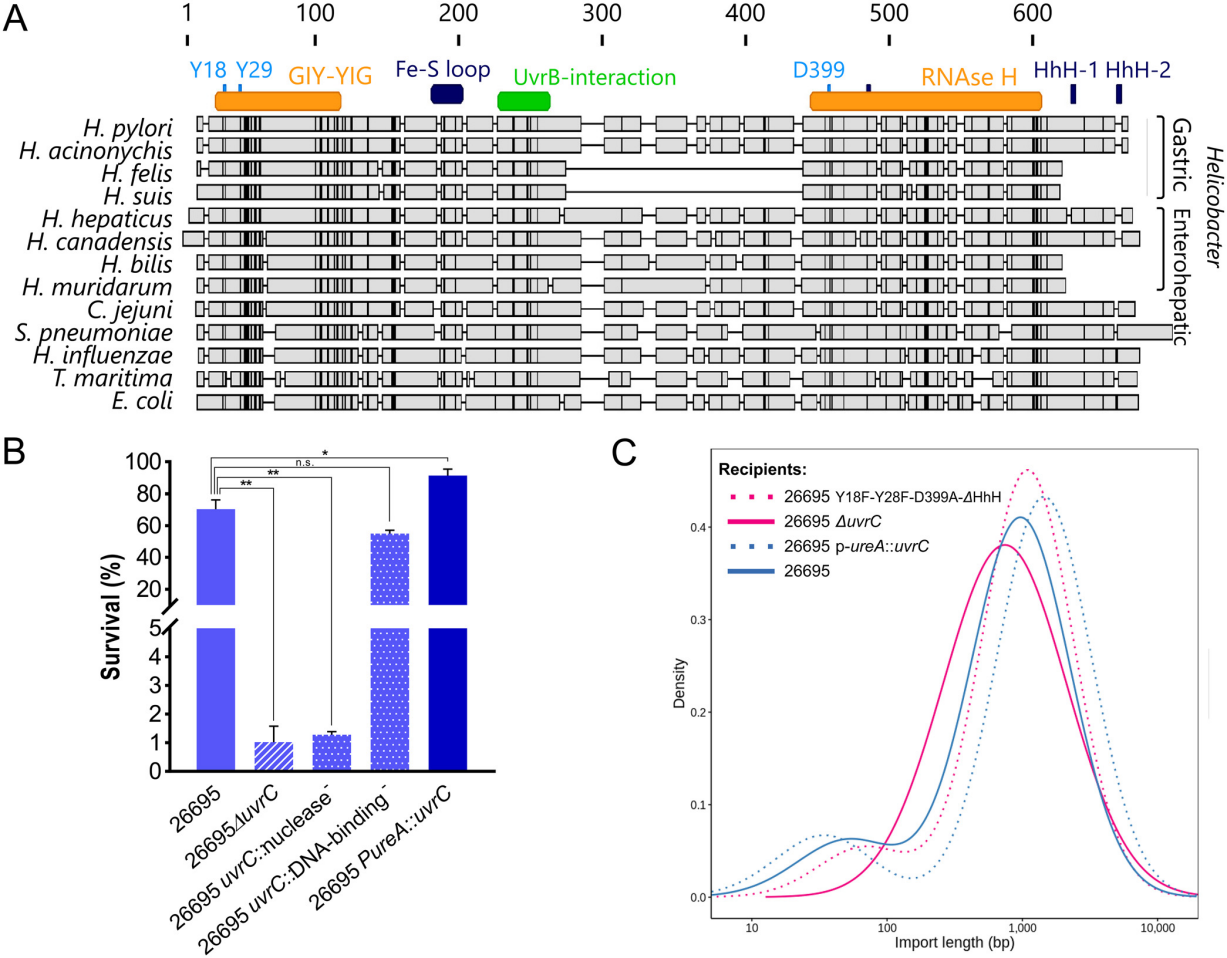
Genetic diversity of UvrC and characterization of its functional domains. (A) Protein sequence alignment of UvrC in H. pylori, other *Helicobacter* species, and selected other bacterial species. Amino acids conserved in all sequences are marked by black lines. Gastric and enterohepatic *Helicobacter* species are indicated by brackets. (B) Susceptibility of different H. pylori *uvrC* mutants to UV irradiation. H. pylori strains were irradiated by UV light, and the percentage of surviving colonies was calculated against that of a control for at least two independent replicates. *, *P* < 0.05; **, *P* < 0.01 (*t* test). Error bars indicate the standard error of the mean. (C) Density estimation of import lengths in the UvrC multidomain mutant and UvrC overexpression strain.

To evaluate the functional relevance of the domains described for UvrC in other species, we built a multiple-sequence alignment of UvrC homologs from *Helicobacter* species, other naturally competent bacteria, and selected other organisms (Fig. 3A). The two highly conserved tyrosine residues of the GIY-YIG catalytic core were also preserved in H. pylori. Modification of the second tyrosine was shown to be sufficient to fully abolish the activity of this domain in T. maritima (52). Similarly, we identified a conserved aspartate residue in the RNase H-like domain that is strictly required for 5′ incision activity (47, 53). In H. pylori, we mutated the catalytic sites in the GIY-YIG domain to phenylalanine and the one in the RNase H-like domain to alanine in order to generate a strain deficient for endonuclease activity. While not associated with any known functional domain, the middle region of UvrC was strikingly absent in the gastric *Helicobacter* species *H. felis* and *H. suis*. Likewise, the DNA-binding region was either truncated, leaving only a single HhH motif (Fig. S2), or completely missing in all the *Helicobacter* species included in this alignment, without any particular correlation with host tropism. In E. coli, both HhH motifs are thought to form a functional unit required for 5′ and/or 3′ incisions (48, 51). Nevertheless, the second motif, but not the first, appeared to be sufficient for binding of ssDNA (51). In H. pylori, we disrupted the single HhH motif by deletion of the GhG hairpin to create a strain putatively deficient for DNA binding.

10.1128/mbio.01811-22.3 FIG S2 Protein structure prediction of the C-terminal DNA-binding domain of the UvrC nuclease using Phyre2. (A) UvrC in Escherichia coli; (B) UvrC in Helicobacter pylori. Hairpin motifs are labeled and colored in red, and the side chains are displayed. Download FIG S2, PDF file, 0.2 MB. Copyright © 2022 Ailloud et al. 2022 Ailloud et al. https://creativecommons.org/licenses/by/4.0/ This content is distributed under the terms of the Creative Commons Attribution 4.0 International license.

The NER pathway is known for its role in repairing UV-induced DNA damage, and thus UV irradiation is typically used to assess its function. Therefore, we tested the UV tolerance of our domain-specific mutants and compared it to those of the wild-type and *uvrC* knockout strains (Fig. 3B). Inactivation of the catalytic residues in the GIY-YIG and RNase H-like domains resulted in a strong defect of UV tolerance, similar to the effect observed in a *uvrC* knockout mutant, indicating that these conserved residues have similar roles in the NER pathway of H. pylori to those described in E. coli and T. maritima. In contrast, mutation of the HhH motif did not markedly increase UV susceptibility compared to that of the wild type, suggesting that the mutated UvrC was still able to perform its function within the NER system. In E. coli, both HhH motifs are required for UV survival (51), and thus the different organization of the domain in H. pylori is likely associated with a specific function. To evaluate the combined effect of these mutations on the bimodal distribution, we constructed a strain including all the domain-specific mutations for the GIY-YIG, RNase H-like and DNA-binding domains and tested it with the pSTT method. The distribution of import lengths was unchanged in this mutant, suggesting that neither the endonucleases nor the putative DNA-binding domain are involved in the integration of microimports mediated via UvrC (Fig. 3C).

Since none of the currently described functional domains of UvrC appeared to have an effect on the bimodal distribution, we investigated whether this protein is involved in a rate-limiting step in the recombination pathway leading to microimports. Indeed, the rarity of microimports compared to macroimports suggests they might be the result of a less efficient pathway. For example, the UvrC protein has been shown to be present at low levels in E. coli (i.e., 10 to 20 copies per cell) (55) and thus the translation rate of UvrC could be a limiting factor for the production of microimports in H. pylori. To test this hypothesis, we created an overexpression mutant in the H. pylori strain 26695 by fusing *uvrC* to the native urease promoter. We confirmed by quantitative PCR (qPCR) that the transcription of *uvrC* was increased more than 10-fold in the mutant compared to the wild-type strain (Text S1). The UV resistance of this UvrC overexpression strain was significantly higher than for the wild type (Fig. 3B). However, the proportion of microimports was not significantly increased by overexpression of *uvrC* (Fig. 3C), indicating that other limiting factors exist beforehand or that the process is easily saturated. Overall, an intrinsic bottleneck affecting the pathway leading to microimports would explain why microimports are rare compared to macroimports. The relative stability of the proportions of microimports between H. pylori strains could also indicate that the rate of microimport integration is constrained by the canonical pathway of HR leading to macroimports.

10.1128/mbio.01811-22.1 TEXT S1 Methods and MIQE guidelines for qPCR assays. Download Text S1, PDF file, 0.2 MB. Copyright © 2022 Ailloud et al. 2022 Ailloud et al. https://creativecommons.org/licenses/by/4.0/ This content is distributed under the terms of the Creative Commons Attribution 4.0 International license.

### Microimports are frequently clustering next to other imports.

**FIG 4:**
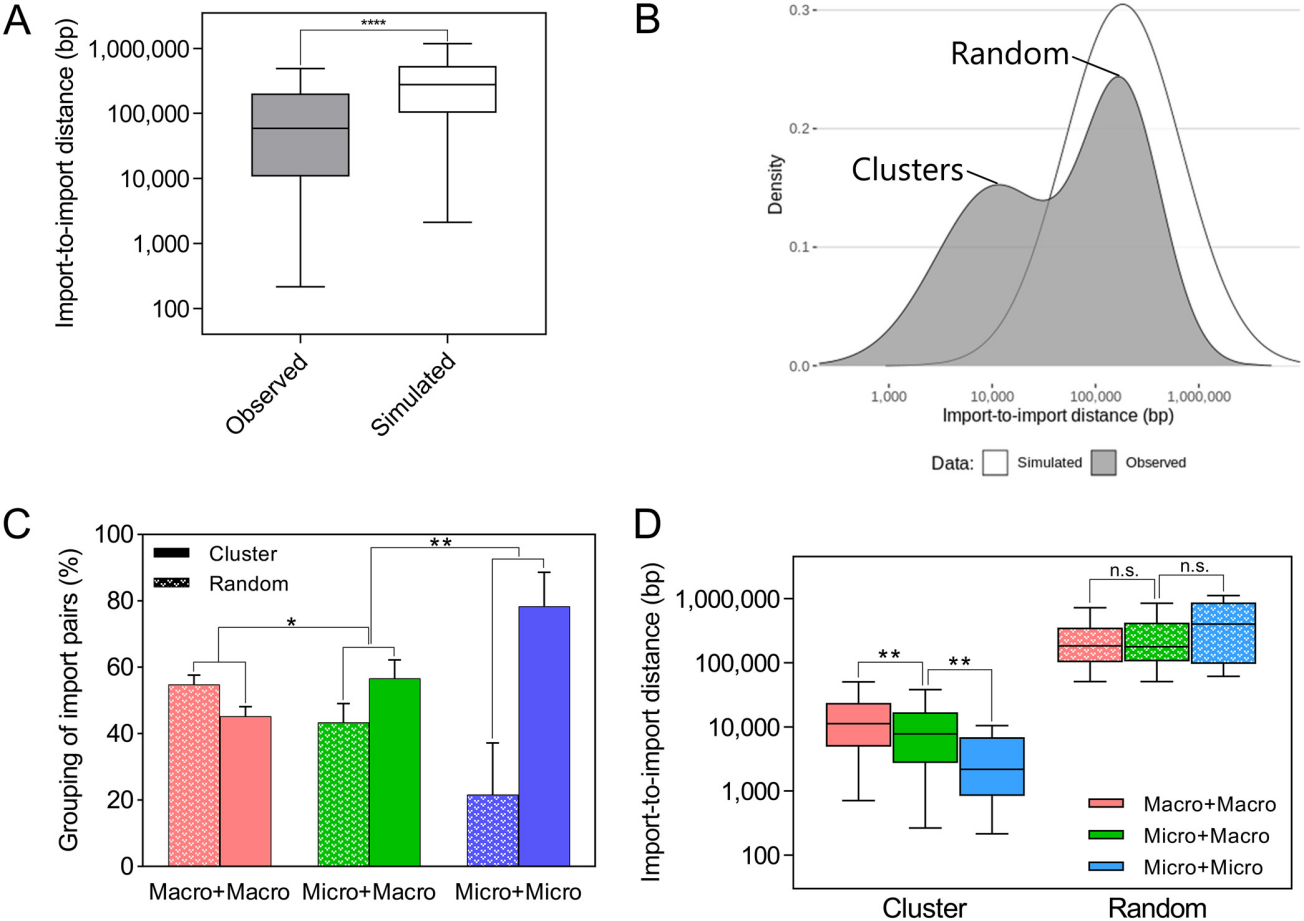
Comparison of import-to-import distances between micro- and macroimports. (A) Box plot of import-to-import distances from observed (experimental) and expected (simulated) data. ***, *P* < 0.0001 (Mann-Whitney U test). Whiskers indicate the minimum and maximal values. Boxes show the interquartile range. The central line represents the median. (B) Density estimation of import-to-import distances for observed and expected data; (C) proportion of clustered versus randomly distributed events according to import length. **, *P* < 0.01 (Fisher’s exact test). Error bars indicate 95% confidence intervals. (D) Variation of import-to-import distances according to import length and clustering. **, *P* < 0.01 (*t* test). Whiskers indicate the minimum and maximal values. Boxes show the interquartile range. The central line represents the median.

Earlier studies have observed that imports seem to be distributed nonrandomly throughout the chromosome of H. pylori (22, 24, 25). Nevertheless, the narrow genomic regions studied by early *in vitro* assays (24, 25) or the uncertainty about donor sequences with *in vivo* data (22) interfered with the quantitative analysis of these events. Here, we calculated the distances between pairs of imports within each transformant in our data set under the assumption that each pair might represent a group of clustered recombination events. In parallel, we simulated distances assuming a random distribution of imports over the chromosome, taking into account the number of imports per transformant observed in our data set. The mean import-to-import distance was significantly different (Mann-Whitney U test, *P* < 0.0001) between the *in vitro* data and the simulation, indicating that imports in H. pylori are in closer proximity than would be expected by chance (Fig. 4A).

Next, we computed the probability density function of our data set and identified two distinct peaks. The first peak had a mean import-to-import distance of 16,390 bp, while the second peak had a mean distance of 273,617 bp. The density of the simulation coincided with the second peak of our data set, suggesting that the first peak represents clustered, nonrandomly distributed, imports (Fig. 4B). In our experimental model, the import-to-import distance is constrained by the definition of clusters of nucleotide polymorphisms (CNPs) used in this study since a pair of distinct CNPs separated by less than 200 bp would actually be considered a single import separated by interspersed sequences of the recipient (ISR) rather than two separate imports (26). ISR appear at low frequency (14% in our data set) and have been associated with the BER pathway (24). Some of the clustered imports we observed here could also result from DNA repair, yet it is unlikely that this mechanism would be responsible for gaps of several thousand bases between imports. As speculated previously, we posit that clustered imports are the outcome of multiple integration events from a single fragment of donor DNA (25). Our previous analyses of the influence of single nucleotide polymorphism (SNP) density on recombination were performed by comparing genuine imports to the rest of the genome (26). Here we compared the SNP density of imports located in a cluster to that of nonimported fragments within the same cluster. Although the difference was minor, the SNP density of imports (*x*¯ = 44 SNPs/kb) was significantly lower (*t* test, *P* < 0.001) than in the rest of the clusters (*x*¯ = 47 SNPs/kb), suggesting that the homology between donor and recipient strains can have a small impact on which segments of the invading DNA fragment preferentially get imported (Fig. S3).

10.1128/mbio.01811-22.4 FIG S3 Nucleotide divergence within import clusters. The density of donor recipient SNPs (SNPs per kilobase) was calculated for all 595 clusters of imports observed in the dataset. The density was calculated separately for (i) the imports located within each cluster (right) and (ii) the nonimported fragments making up the rest of the cluster (left) (*P* < 0.001, *t* test). The data are represented as a box plot with the probability density function displayed alongside it. Download FIG S3, PDF file, 0.03 MB. Copyright © 2022 Ailloud et al. 2022 Ailloud et al. https://creativecommons.org/licenses/by/4.0/ This content is distributed under the terms of the Creative Commons Attribution 4.0 International license.

In this analysis, import clusters can consist of both micro- and macroimports. The length and the mechanism leading to the incorporation of imports might influence the formation of clusters. Consequently, we compared the composition of clustered versus nonclustered imports. Pairs containing one macro- and one microimport were more frequently clustered than those containing only macroimports (Fisher’s exact test, *P* < 0.01). Similarly, pairs consisting of microimports only were more frequently grouped than mixed pairs (Fig. 4C). Furthermore, the import-to-import distance within a cluster also decreased (*t* test, *P* < 0.01) according to the number of microimports within the pair (Fig. 4D). Altogether, this analysis suggests that transforming DNA can be integrated in a clustered manner during natural transformation in H. pylori and that the emergence of an import cluster favors the incorporation of microimports.

## DISCUSSION

Homologous recombination by natural transformation is a key mechanism for H. pylori to quickly acquire diversity during mixed infections (6, 56, 57). The length of imports follows a bimodal distribution, suggesting the existence of two distinct mechanisms for integration of exogenous DNA (26). Here, we improved our whole-genome transformation assay in order to explore the factors influencing the import length in H. pylori. Bimodality was conserved between different genetic backgrounds and in strains from different phylogeographic populations. The deviations of the mean size of imports and of the proportion of microimports can mostly be attributed to experimental variation. Notably, the recipient-donor pairs display different levels of nucleotide divergence, which affects the calculation of the median import length. Interestingly, strain-specific competence patterns have been observed in H. pylori, including in strains 26695 and J99 (38, 58), but do not appear to shape the distribution of import length.

Of the four proteins involved in the NER pathway, only UvrC was associated with promoting the integration of microimports. In model organisms, the UvrC protein has only been functionally characterized in the context of DNA damage repair via the NER pathway. In H. pylori, NER mutants are all strongly impaired for DNA repair, indicating that all enzymes are required for the normal operation of the pathway (40). In particular, the UvrA_2_B_2_ complex is required to recognize DNA damage, with UvrC catalyzing the incision of the lesion and UvrD displacing the damaged segment (32, 42). Consequently, the role of UvrC in the incorporation of the microimports does not appear to be related to the typical operation of the NER pathway.

In three different genetic backgrounds, the deletion of *uvrC* led to a shift from a bimodal to a unimodal distribution of import lengths, which was subsequently restored by functional complementation. These results suggest that UvrC is involved in a specific mechanism responsible for the peak of microimports, while the peak of macroimports is the product of the canonical HR pathway (6, 32). We previously reported, using a transformation assay limited to a single locus (*rpoB*), that UvrC globally limits the import length in H. pylori (40): i.e., that imports were longer in *uvrC* mutants. This early study was performed following the assumption that imports in H. pylori followed a unimodal distribution with a single mean import length. As a result of this, the overall import length appeared to increase in the *uvrC* knockout background. Under the bimodal distribution model (26), our new whole-genome and functional data show here that the deletion of *uvrC* only affects the frequency of microimports, while the length of macroimports remains unchanged.

This has important implications with respect to the role of UvrC. With the exception of *recA*, no other H. pylori genes have been shown to be strictly essential for HR in H. pylori, suggesting the existence of alternative HR pathways (6). Our study represents the first experimental evidence that other pathways indeed exist in H. pylori and demonstrates that UvrC does not participate in the integration of every import but instead contributes to a specific pathway generating microimports. We note that short fragments appear to still be integrated at low frequency via the canonical pathway, supporting the hypothesis that the boundaries of imports during HR are determined by a stochastic process in H. pylori (26). Such a process has also been described in S. pneumoniae and has been proposed to be connected to the stochastic resolution of heteroduplex intermediates (59). Therefore, UvrC may be involved in a process modulating the canonical HR pathway, specifically increasing the probability of integration of short fragments and leading to a peak of microimports, rather than a completely independent pathway of HR. This is supported by the fact that the nuclease catalytic sites of UvrC did not contribute to the peak of microimports. Above all, this implies that UvrC is not involved in the cleavage of incoming DNA during synapsis nor in the incision of recombination intermediates during resolution. In H. pylori, both catalytic sites of UvrC are very similar to the domains found in homologs from other organisms and naturally competent bacteria. However, the organization of the DNA-binding domain appears to be unique to *Helicobacter* species. Notably, a single HhH motif is found in H. pylori, while two have been described in other organisms and are considered necessary for the proper function of UvrC (48, 50, 53, 60). Deletion of the hairpin within this single motif did not appear to impair the normal activity of UvrC as reflected by the absence of an effect on UV susceptibility. In some of the gastric and enterohepatic *Helicobacter* spp. considered in this study, the entire DNA-binding domain is absent, indicating it was no longer under purifying selection in those species. Therefore, we cannot conclude whether this domain is actually functional in H. pylori. Historically, UvrC has been a challenging protein to study, and a full-length crystal structure is still not available. Different regions in UvrC could also be important for DNA binding. A patch of conserved residues located in the RNase H-like domain has been associated with creating a DNA-protein interface essential for both 3′ and 5′ incisions (53). Recently, an iron-sulfur cluster loop motif was identified downstream of the GIY-YIG domain and was shown to participate in the formation of UvrC-dsDNA complexes when bound to its [4Fe4S] cofactor (61). In model organisms, activity of UvrC separated from the NER pathway has been described *in vitro*. Notably, it is able to form a stable complex with DNA in Bacillus caldotenax (62) and to participate in DNA charge transport in E. coli (61). A tetrameric form of UvrC, with the ability to bind dsDNA and a similar affinity for intact or damaged DNA, has also been described in E. coli (63, 64). Considering the atypical domain organization of UvrC in *Helicobacter* spp. and H. pylori, it is probable that UvrC diverged functionally compared to the UvrC characterized in model organisms.

In H. pylori, the tendency of exogenous DNA to integrate contiguously has been observed several times in earlier studies (22, 24, 25). Within the experimental settings of the *in vitro* transformation assay, we showed that a large proportion of imports were nonrandomly distributed along the chromosome of H. pylori and occurred in clusters of 16,390 bp on average. Interestingly, microimports were significantly more frequent within clusters than macroimports, and clusters consisting only of microimports were shorter than clusters containing at least one macroimport. The mean size of the import clusters observed in this study is not compatible with the generation of clusters via DNA repair of short patches; therefore, we speculate that import clusters are mainly the result of multiple integration events occurring from a common fragment of DNA. A clustering of imports has also been noted in S. pneumoniae, but was not associated with imports of a specific length (59). In H. influenzae, clusters with a mean length of 19,500 bp were observed, which is similar to the length obtained in this study (12). Nonetheless, the integration of multiple imports from a single donor DNA fragment has not been directly observed yet and needs to be further characterized *in vitro*. Moreover, the conditions that influence whether one or multiple imports gets integrated remain to be determined.

**FIG 5:**
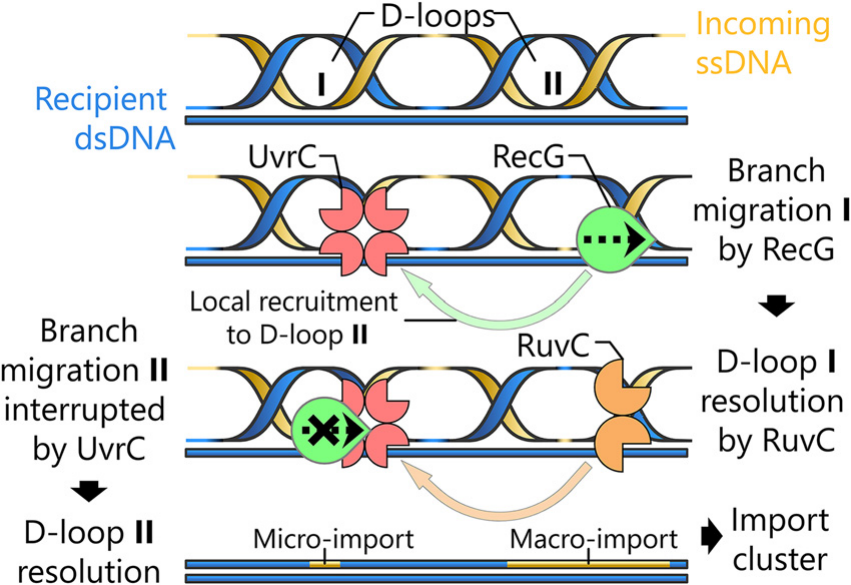
Hypothetical model for the clustered integration of microimports during natural transformation in H. pylori. Following strand exchange, multiple displacement loops (D-loops) can occur along the same fragment of incoming DNA during synapsis. Branch migration, driven by RecG, can either proceed normally or be sterically hindered by multimers of UvrC bound to heteroduplex junctions. Subsequent resolution by RuvC would generate macroimports after normal branch migration, whereas interference by UvrC would result in microimports.

Considering the results presented in this study, we propose a hypothetical model to explain the bimodal distribution of import length in H. pylori (Fig. 5). Following DNA uptake by the ComB system and RecA-mediated strand invasion, several adjacent synapsis events can be initiated from a single fragment of donor DNA. Stochastically, the tetrameric form of UvrC is able to bind D-loop intermediates, which could subsequently lead to steric hindrance of branch migration and the integration of microimports. While the affinity of HR factors for D-loops, such as the RecG helicase or the RuvC resolvase, is likely reduced in the presence of UvrC, the formation of multiple adjacent strand invasions from an individual fragment of transforming DNA might promote the branch migration of such D-loops due to local concentration effects. Indeed, the proximity of several DNA intermediates could favor rebinding of UvrC-bound D-loops when HR factors are dissociating from a neighboring D-loop following its resolution.

Nonetheless, the biological function and evolutionary logic behind microimports in H. pylori remain to be determined. Transformation in H. pylori is highly efficient (9, 10), albeit variable according to genetic background and environmental conditions (58, 65, 66). It has been shown to promote chronic infection in a mouse model (67) and has the potential to generate phenotypically diverse populations in the human stomach (68, 69). The bimodal distribution of import lengths is likely conserved across the species, suggesting it might provide a fitness advantage. In S. pneumoniae, aberrant behavior of the MMR system is believed to be responsible for the integration of longer fragments (27). Such HR process could be advantageous by inducing more phenotypic changes under immune-driven selection (70) or permitting the recombination of more divergent DNA in biofilms (71). In contrast, an atypical HR mechanism mediated by UvrC seems to lead to the integration of shorter fragments in H. pylori. Considering the genome size of H. pylori and the high divergence between strains, recombination during mixed infections has the potential to radically change the genomic landscape of a H. pylori population within the stomach niche. A dedicated system in charge of importing only a limited amount of genetic diversity might have emerged to allow a more fine-tuned evolution. Phenotypes related to antibiotic resistance (72) or adhesion (73, 74) have been associated with complex allelic variation of individual genes. In such a context, recombination of microimports may allow the acquisition of single mutations within a gene and thus promote diversity by increasing the number of possible new allelic variants generated at the scale of the population. In the absence of microimports, recombination of macroimports is more likely to replace a large fraction of a given gene and thus limiting the potential allelic combinations in the population. Because H. pylori is uniquely adapted to its host and restricted the stomach niche, global acquisition of deleterious genotypes could quickly lead to fatal population bottlenecks during hard selective sweeps. Indeed, bottlenecks are believed to occur frequently during H. pylori infections (18, 68), indicating that maintaining population integrity is likely a central part of the H. pylori infectious cycle. In S. pneumoniae, it was determined recently that transformation efficiency is limited to approximately 50% of the population, which is thought to be a fail-safe mechanism to preserve original copies of the genome (75). In order to fully understand the role of natural transformation in the lifestyle of H. pylori, future *in vitro* experimental evolution analysis and *in vivo* experimental infection studies will need to consider the contribution of both micro- and macroimports to the genetic diversity of the species.

To determine the validity of the hypothetical model proposed in this study, further *in vitro* characterization of the function of UvrC in H. pylori is required. In particular, characterizing the way UvrC interacts with HR intermediates will be central to understand how this enzyme influences the import length. Finally, a full-length crystal structure would also contribute to understanding the impact of the interspecies variability observed in the middle and C-terminal regions of UvrC.

## MATERIALS AND METHODS

### Bacterial strains and culture conditions.

Bacterial strains used in this study are listed in Table S1 in the supplemental material. H. pylori strains were cultured on blood agar plates containing 10% horse blood and antibiotics (vancomycin [10 mg/L], polymyxin B [3.2 mg/L], amphotericin B [4 mg/L], and trimethoprim [5 mg/L]) or in brain heart infusion (BHI) liquid medium with yeast extract (2.5 g/L), 10% heat-inactivated horse serum, and antibiotics, as described above. Cultivation was performed at 37°C in a microaerobic atmosphere using airtight jars (Oxoid, Wesel, Germany) and Anaerocult C gas-generating bags (Merck, Darmstadt, Germany). E. coli strains were cultured on LB agar plates or in LB liquid medium with appropriate selective antibiotics at the following concentrations: ampicillin, 200 μg/mL; kanamycin, 20 μg/mL; or chloramphenicol, 20 μg/mL.

10.1128/mbio.01811-22.5 TABLE S1 Bacterial strains used in this study. Download Table S1, PDF file, 0.04 MB. Copyright © 2022 Ailloud et al. 2022 Ailloud et al. https://creativecommons.org/licenses/by/4.0/ This content is distributed under the terms of the Creative Commons Attribution 4.0 International license.

### DNA and RNA isolation.

Genomic DNA (gDNA) was extracted from bacteria grown on blood agar plates for 24 h with the Genomic-tip 100/G kit or QIAamp DNA minikit (Qiagen, Hilden, Germany) following the manufacturer’s protocol. The isolated gDNA was dissolved in EB buffer from the same kit. RNA was extracted from bacteria grown in liquid cultures as described in Text S1.

### Construction of markerless *uvrC* deletion mutants.

The oligonucleotides and plasmids used in this study are described in Tables S2 and S3, respectively. Markerless deletion mutants were generated through multiplex genome editing by natural transformation (MuGENT) (76), using a protocol adapted to H. pylori (26). Briefly, homology arms of 1 kb flanking *uvrC* (*hp0821*) were amplified from H. pylori strain J99 or N6. Oligonucleotides were designed to introduce a restriction site 5′ of the upstream fragment and 3′ of the downstream fragment, as well as matching overlap sequences 3′ of the upstream fragment and 5′ of the downstream fragment. Upstream and downstream fragments were fused by overlap PCR (77) and ligated into the multiple-cloning site of pUC19 using the restriction sites. The resulting 2-kb insert was amplified by PCR to create the unselected PCR product. Cotransformation of H. pylori was performed using a mixture of 1.5 μg of the unselected PCR product and 500 ng of a selected PCR product, consisting of the *rdxA* locus disrupted by an *aphA3* cassette, conferring kanamycin resistance. Transformants were selected on blood agar plates containing kanamycin. After DNA extraction, correct deletion of the *uvrC* locus was assessed by PCR and Sanger sequencing.

10.1128/mbio.01811-22.6 TABLE S2 Primers used in this study. Download Table S2, PDF file, 0.04 MB. Copyright © 2022 Ailloud et al. 2022 Ailloud et al. https://creativecommons.org/licenses/by/4.0/ This content is distributed under the terms of the Creative Commons Attribution 4.0 International license.

10.1128/mbio.01811-22.7 TABLE S3 Plasmids used this study. Download Table S3, PDF file, 0.03 MB. Copyright © 2022 Ailloud et al. 2022 Ailloud et al. https://creativecommons.org/licenses/by/4.0/ This content is distributed under the terms of the Creative Commons Attribution 4.0 International license.

### Site-directed mutagenesis of the *uvrC* gene.

Selected nucleotides of the *uvrC* gene in H. pylori strain 26695 were modified by site-directed mutagenesis. The complete conservation of each targeted nucleotide in H. pylori was verified in >500 genomes from the PATRIC database (78). First, the *uvrC* gene was amplified with specific primers introducing restriction sites at the 3′ and 5′ ends of the gene. The amplicon was ligated into pUC19 using Quick Ligase (NEB, Frankfurt am Main, Germany) and used to transform E. coli MC1061. The resultant plasmid was subjected to inverse PCR, where two adjacent primers were used to generate a linear amplified copy of the plasmid, with the mutations introduced by the forward primer. Right after the inverse PCR, 1 μL of DpnI was added (30 min at 37°C) to remove the methylated plasmid template from the nonmethylated linearized amplicon. The amplicon was then religated and used to transform E. coli MC1061. H. pylori was cotransformed with the resultant plasmid and an *rdxA*::*aphA3* PCR product. Sequences of the modified plasmid and *uvrC* gene of H. pylori were checked by Sanger sequencing.

### Construction of *uvrC* overexpression and complemented strains.

The complete *uvrC* sequence (*hp0821*) was amplified using specific oligonucleotides introducing restriction sites at the 5′ and 3′ ends of the gene. The amplicon was ligated with a digested pADC/*aphA3* suicide plasmid, as described before (79), and then transformed into E. coli MC1061. Approximately 1 μg of the resultant plasmid was used to transform H. pylori, where the *uvrC* gene integrated within the urease locus under the control of the strong *ureA* promoter. The wild-type 26695 strain was transformed to create an overexpression strain, and the 26695 *ΔuvrC* knockout strain was transformed to create a complemented strain.

### Quantitative PCR.

One microgram of RNA was used for cDNA synthesis using the SuperScript III reverse transcriptase (Thermo Fisher Scientific, Darmstadt, Germany), as described before (80). Quantitative PCR (qPCR) was performed in a Bio-Rad CFX96 system with gene-specific primers (Text S1) and SYBR green master mix (Qiagen, Hilden, Germany). Standard curves were produced, and samples were run as technical triplicates. For quantitative comparisons, samples were normalized to an internal 16S rRNA control qPCR. The reaction conditions complying with the MIQE guidelines are available in Text S1.

### Transformation assay of H. pylori.

In order to quantitate import lengths in different H. pylori wild-type strains and mutants, H. pylori was transformed using a modified version of the previously described short-term transformation (STT) protocol (26). A spot from an overnight culture of a recipient H. pylori strain was transferred to a fresh nonselective blood agar plate. After a 5 h of incubation, a mixture of (i) 1,000 ng of gDNA extracted from the donor H. pylori strain and (ii) 100 ng of gDNA extracted from the same donor background with an *rdxA* locus disrupted by a chloramphenicol acetyltransferase (CAT) cassette was added to the spot. After 24 h of incubation, the spot was transferred to three selective blood agar plates containing chloramphenicol. Transformants were picked after 3 to 4 days of incubation. The number of replicates performed for each strain and the number of clones used for each analysis are indicated in Table S4.

10.1128/mbio.01811-22.8 TABLE S4 Clones and replicates data for the STT experiment. Download Table S4, PDF file, 0.03 MB. Copyright © 2022 Ailloud et al. 2022 Ailloud et al. https://creativecommons.org/licenses/by/4.0/ This content is distributed under the terms of the Creative Commons Attribution 4.0 International license.

### Genome sequencing and assembly.

Whole-genome sequencing was performed using Illumina MiSeq technology. Libraries were constructed with Nextera XT DNA sample preparation kits (Illumina) according to the manufacturer’s protocol. Libraries were quantified with a Qubit 3.0 fluorometer and the Qubit dsDNA high-sensitivity assay kit (Invitrogen). Average library sizes were determined with a TapeStation 4200 and the high-sensitivity D5000 kit (Agilent). Libraries were manually normalized to 4 nM and multiplexed in groups of 50 samples using the Nextera XT Index kit v2 (Illumina). The resulting pools were diluted to 10 pM and sequenced using the MiSeq reagent kit v3 (600 cycles, 300-bp paired end).

Paired-end reads were trimmed with Trimmomatic 0.36 (81) using the Nextera adapter sequences and the following parameters: LEADING:20 TRAILING:20 SLIDINGWINDOW:4:15 MINLEN:100. Trimmed paired-end reads were assembled using SPAdes 3.9.0 (82) with the –careful and –only-assembler parameters. Quality of the assemblies was assessed with QUAST (83).

### Chromosomal import pattern analysis.

Contigs from transformants were mapped onto the reference genome of their corresponding recipient strain—26695 (4), J99 (84), N6 (85), or BCM300 (86)—using BWA-MEM 0.7.12 (87). Pileup files were generated using samtools 1.10 with the -B parameter (88). Polymorphisms were called using VarScan 2.4 (89) with the following parameters: –min-coverage 1–min-reads2 1 –min-freq-for-hom 1 –strand-filter 0.

A region of approximately 150,000 bp around the insertion site of the resistance cassette was excluded from the analysis to prevent the influence of “selected imports” with an abnormal import length (26). Based on the differences between recipient and donor strains, variants were filtered to retain only the polymorphisms resulting from recombination with the donor strain gDNA. Duplicated and repeat-rich regions of the genome of H. pylori were also excluded from the analysis in order to avoid mapping artifacts. This includes copies of the 16S, 23S, and 5S rRNA (4), the *futA* and *futB* paralogs (90), *cagY* (91), and the DUF874 family proteins (92). The recipient strains used in this study are “laboratory strains,” which have been passaged repeatedly and can diverge slightly over time. To account for this effect, variants observed in more than 50% of the transformants were considered part of the recipient strain diversity and excluded from the analysis. Imports were delineated based on parameters adapted from previously described rules (26). Clusters of nucleotide polymorphisms (CNPs) consist of at least two polymorphisms separated by stretches smaller than 200 bp, based on the probability distribution of the length of import flanking regions previously calculated (21, 26). CNPs can be interrupted by interspersed sequences of the recipient (ISR) with a maximal length of 200 bp. The definition of single polymorphism imports (SPIs) was relaxed, and they now contain one polymorphism flanked by at least 20 bp of sequence identical between donor and recipient and located more than 5 bp away from artifactual polymorphisms not found in the donor strain (e.g., sequencing errors, spontaneous mutations, etc.). Imports occurring identically in multiple transformants were considered artifactual and discarded from the analysis.

Minimal import lengths are calculated from the first to the last polymorphism of the recombination tract, while maximal import lengths are calculated including the stretches of donor-recipient identical sequence flanking the recombined polymorphisms. For subsequent analysis, the median import lengths were used, which correspond to the average between the minimal and maximal import lengths (26). After log transformation, the distribution of import lengths was analyzed with the mclust R package for density estimation based on finite normal-mixture modeling via an expectation maximization algorithm (93). The number of components was estimated via a likelihood ratio test. Parameters of the mixture distribution were inferred from a nonparametric resampling bootstrap approach with 1,000 iterations. To generate plots, data points were simulated (10,000 iterations) from the mixture models estimated by mclust and plotted with the ggplot2 R package (94).

### UV assay.

UV sensitivity assays were performed as published previously (40). Briefly, a fresh culture of H. pylori was resuspended in BHI medium. After the absorbance was measured, the cell suspension was diluted and plated on blood agar plates. For each dilution, three plates were used as control and three plates were exposed to UV for 2 s (Osram HNS 30-W OFR, UV-C, 254 nm, 12 W) at a distance of 40 cm (≅100 J/m^2^). Plates were incubated for 72 h, and colonies were counted to calculate the survival rate.

### Import linkage analysis.

The probability distribution of import length was estimated using all the available data using the mclust R package as described previously. The maximum length of the first component of the distribution (i.e., the peak of microimports) was used as a threshold to classify micro- and macroimports. Distances between each pair of consecutive imports, accounting for a circular chromosome, were calculated for each transformant. Distances overlapping the discarded region surrounding the resistance cassette were excluded from the analysis in order to avoid artificially inflated distances.

Simulation of expected distances between imports, under the assumption that imports are fully independent, was performed by random sampling based on the number of imports per clone we obtained in our experimental data set (e.g., if five imports were observed in a given transformant, five imports were simulated randomly around the chromosome). The probability distribution of the log-transformed expected and observed distances between imports was obtained using the mclust R package. The mixture model was chosen using the Bayesian information criterion for the observed distances, while it was constrained to one component for the expected distances. The maximum distance of the first peak obtained with the distribution of the observed distances was used as a threshold to classify clustered and randomly distributed imports.

### Data availability.

Raw sequencing data generated in this study were deposited in the NCBI SRA database under BioProject accession no. PRJNA765905.
