# Supplementary figures and images for: The Helicobacter pylori UvrC Nuclease Is Essential for Chromosomal Microimports after Natural Transformation
Source: mBio. 2022 Jul 25;13(4):e01811-22. doi: 10.1128/mbio.01811-22 (PMC9426483; doi:10.1128/mbio.01811-22)

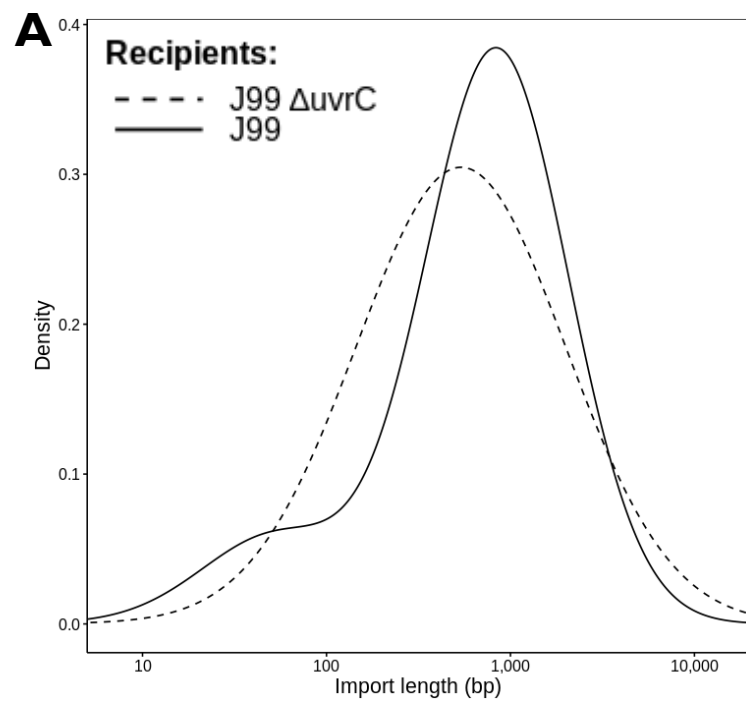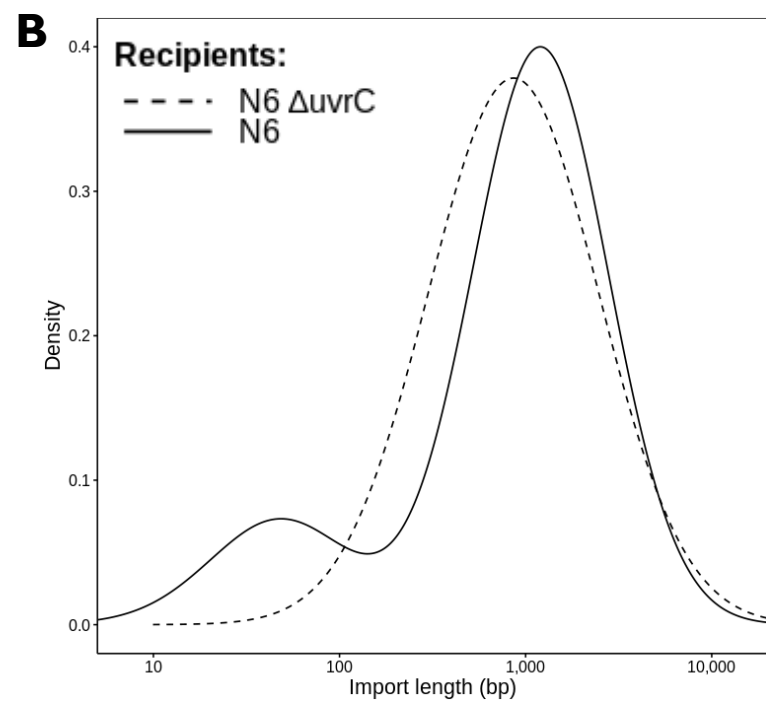

Supplement: FIG S1 [file mbio.01811-22-s0001.pdf]

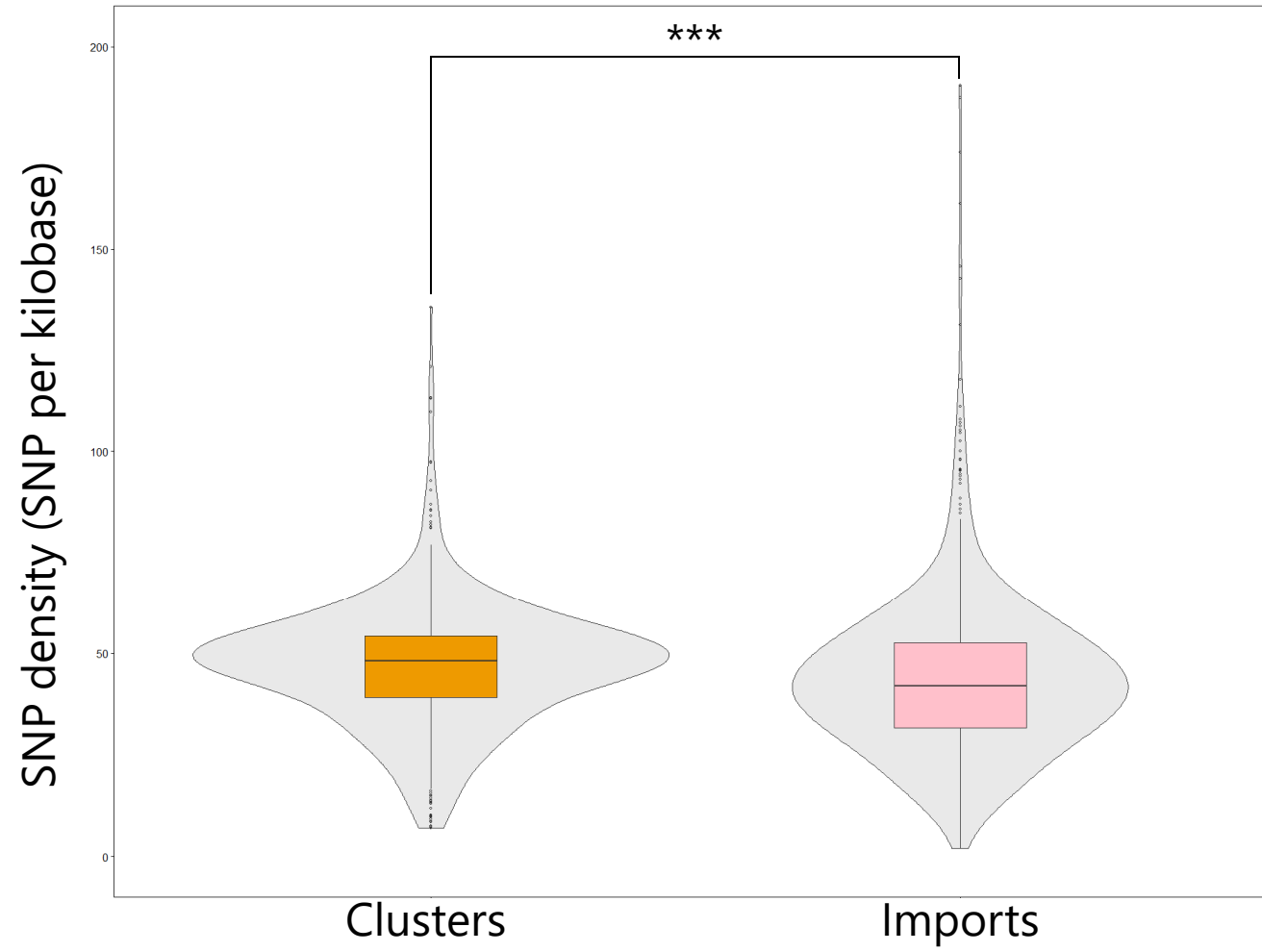

Supplement: FIG S3 [file mbio.01811-22-s0003.pdf]
