# Supplementary material for: The Helicobacter pylori UvrC Nuclease Is Essential for Chromosomal Microimports after Natural Transformation
Source: mBio. 2022 Jul 25;13(4):e01811-22. doi: 10.1128/mbio.01811-22 (PMC9426483; doi:10.1128/mbio.01811-22)
Supplement: FIG S2 [file mbio.01811-22-s0002.pdf]

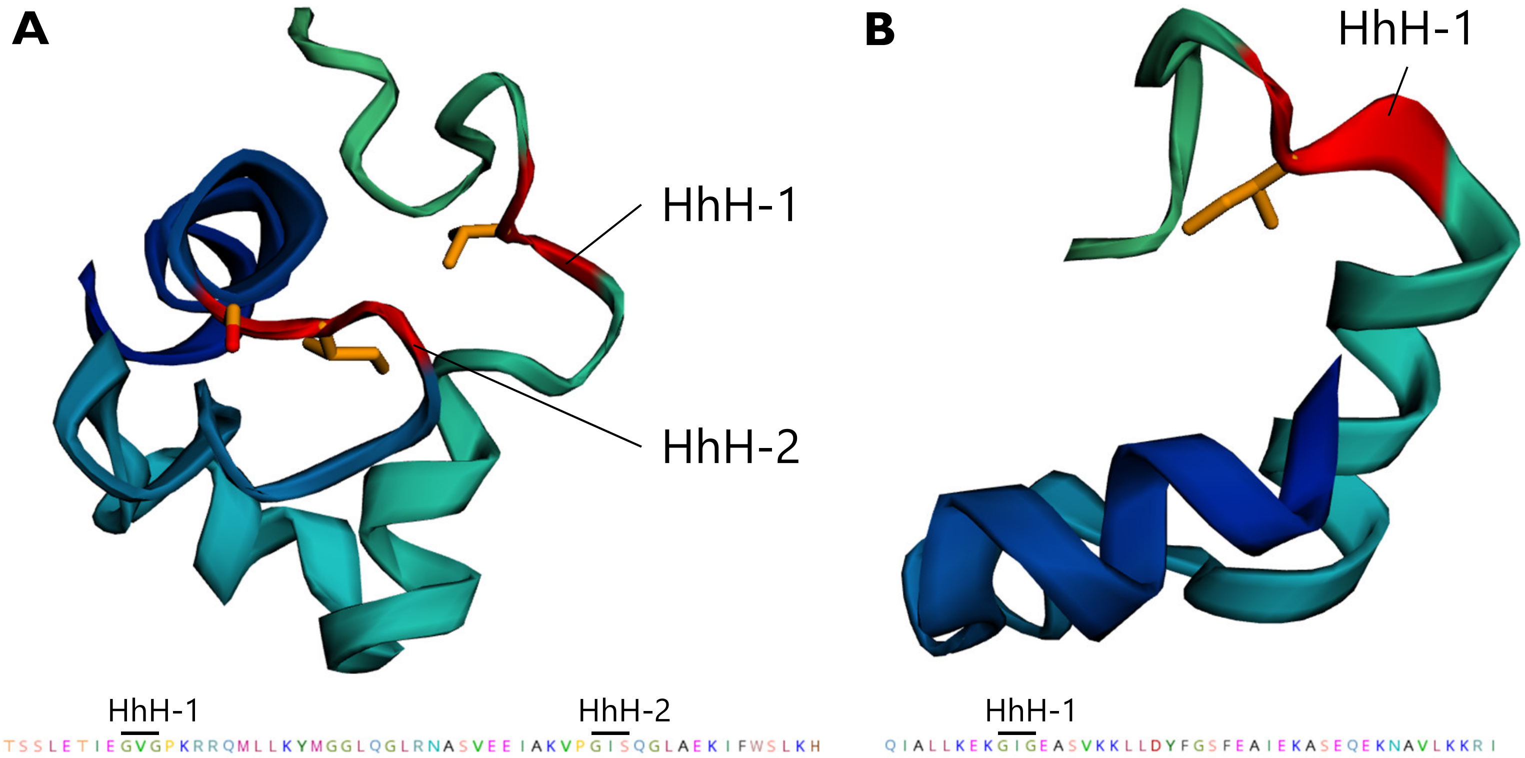
A
HhH-1
HhH-2
HhH-1 HhH-2
TSSLETIEGVGPKRRQMLLKYMGGLQGLRNASVEEIAKVPGISQGLAEKIFWSLKH
B
HhH-1
HhH-1
QIALLKEKGIGEASVKKLLDYFGSFEAIEKASEQEKNAVLKKRI
