# Supplementary material for: The Helicobacter pylori UvrC Nuclease Is Essential for Chromosomal Microimports after Natural Transformation
Source: mBio. 2022 Jul 25;13(4):e01811-22. doi: 10.1128/mbio.01811-22 (PMC9426483; doi:10.1128/mbio.01811-22)

MIQE guidelines for qPCR assays

qPCR was performed as in (Estibariz et al 2019) with minor modifications. All primers used are listed below.

1. Samples

Helicobacter pylori strains were grown in liquid medium as described in the methods section of the manuscript. Bacteria were grown under microaerobic conditions in airtight jars (Oxoid, Wesel, Germany) with Anaerocult C gas-producing bags (Merck, Darmstadt, Germany), incubated with shaking at 140 rpm and 37°C. For RNA extraction, 5 ml of the cultures were pelleted (4°C, 6000 x g, 3 min), snap-frozen in liquid nitrogen and stored at -80 °C.

2. RNA isolation

Bacterial pellets were disrupted using a FastPrep® FP120 Cell Disrupter (Thermo Savant) using Lysing Matrix B tubes (2 ml) containing 0.1 mm silica beads (MP Biomedicals, Eschwege, Germany). The RNeasy kit (QIAGEN, Hilden, Germany) was used to isolate bacterial RNA. Two DNase treatments were performed: i) on-column treatment with DNase I and ii) using the Ambion Turbo DNA-free™ Kit (Ambion, Kaufungen, Germany) as described by the manufacturer. Absence of DNA contamination in the isolated RNA was checked by PCR with primers targeting housekeeping *efp* gene. RNA concentration was measured with the NanoDrop 2000 spectrophotometer (Peqlab Biotechnologies).

3. Reverse transcription

One µg of RNA was transcribed into cDNA using the SuperScript™ III Reverse Transcriptase (Thermo Fisher Scientific, Darmstadt, Germany). The protocol is as followed:

Step 1: RNA-Primer Mix

Component	Volume
Total RNA	1 µg
Random primers (Invitrogen) 3µg/µl (1:60)	2 µl
RNAse-free water	Addition up to 27 µl

Five minutes incubation at 65°C followed by 1 minute on ice.

Step 2: Setting up the cDNA synthesis-Mix

Component	Volume (1x)
5x First-Strand Buffer ($MgCl_2$) (Invitrogen)	8 µl
DTT (0,1 M) (Invitrogen)	2 µl
dNTP (10 mM)	1 µl
RNaseOUT (40 U/µl) (Invitrogen)	1 µl
SuperScript III RT (200 U/µl) (Invitrogen)	1 µl

Addition of the 13 µl mix to the RNA-primer mix (27 µl) for a total volume of 40 µl.
Five minutes incubation at room temperature (25°C).
Inactivation was performed incubating 15 minutes at 70°C.

Step 3: Checking cDNA synthesis via PCR

Correct synthesis of cDNA was checked via PCR using primers for the *efp* housekeeping gene. cDNA was stored at -20°C.

4. Target

The genome of *H. pylori* 26695 is publically available on the NCBI Database and published previously (Tomb, et al 1997).

5. Primers

Primer name	Sequence	Direction	Amplicon length	Gene
qPCR_UvrC_for	ATGGTCAAACAGATCGCTTT	for	186 bp	*uvrC*
qPCR_UvrC_rev	TCGTGTGATTAAAGGGATAGG	rev		
16S_RT1	TTACTAGCGATTCCAGCTTC	for	272 bp	16S
16S_RT2	TGAGATGTTGGGTTAAGTCC	rev		
HPefpF02	GGGCTTGAAAATTGAATTGGGCGG	for	501 bp	*efp*
HPefpR01	GTATTGACTTTAATGATCTCACCC	rev		

6. qPCR cycle

Different concentrations of standards for all genes were prepared in ultrapure water (2 pg/µl, 0.2 pg/µl, 0.02 pg/µl, 2 fg/µl, 0.2 fg/µl). Gene specific primers and SYBR Green Master Mix (QIAGEN, Hilden, Germany) were used for qPCR. Reactions were prepared as listed here:

Component	Volume (1x)
SYBR Green Master Mix	10 µl
Primer forward	1 µl
Primer reverse	1 µl
RNAse-free water	7.5 µl
cDNA	0.5 µl

qPCR protocol run:

1: 95.0°C for 15:00 min
2: 95.0°C for 0:15 min
3: X for 0:30 min (X determine the temperature of each primer combination)
4: 72.0°C for 0:30 min
Plate Read
5: GOTO 2, 39 more times (40 cycles in total)
6: Melt Curve 60.0°C to 95.0°C: Increment 0.5°C 0:05

7. Data analysis

qPCR reactions were run in a BioRad CFX96 system that defines the thresholds, Cq and Sq values. Samples were run as technical triplicates. Calculations of the Cq values and standard curves were automatically determined by the CFX software taking into account the standards included in each qPCR run. Negative controls were prepared with nuclease-free water. All samples were normalized to an internal 16S rRNA control qPCR also run in technical triplicates. The mean Sq values of the technical triplicates of the control (16S) were normalized to the wild-type strain (set to 1) to create correction factors. Then, the mean Sq values of the target gene was corrected with the calculated correction factor for each strain.

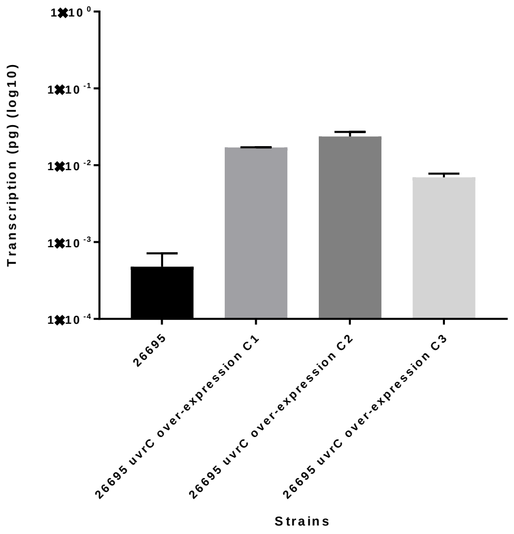

Supplement: TEXT S1 [file mbio.01811-22-s0004.pdf]
