# Supplementary material for: The Helicobacter pylori UvrC Nuclease Is Essential for Chromosomal Microimports after Natural Transformation
Source: mBio. 2022 Jul 25;13(4):e01811-22. doi: 10.1128/mbio.01811-22 (PMC9426483; doi:10.1128/mbio.01811-22)
Supplement: TABLE S1 [file mbio.01811-22-s0005.pdf]

Strain	Genotype	Source
Escherichia coli		
MC1061	*araD139* , Δ(*ara* , *leu*)7697, Δ*lacX74* , *galU* $^{-}$, *galK* $^{-}$, *hsr* $^{-}$, *hsm* $^{-}$, *strA*	Casadaban et al. 1980
Helicobacter pylori		
26695	Wild-type	Tomb et al. 1997
J99	Wild-type	Alm et al. 1999
N6	Wild-type	Ferrero et al. 1992
BCM300	Wild-type	Malfertheiner et al. 2018 (The Lancet Gastro)
BAC339	J99-R3*rdxA* ::*cat*, *flaA* ::*aphA3*	Bubendorfer et al. 2016
BAC569	26695*rdxA* ::*cat*	Bubendorfer et al. 2016
SBac70	26695Δ*uvrA, rdxA::aphA3*	This study
BAC231	26695Δ*uvrB* ::*aphA3*	Moccia et al. 2012
BAC574	26695Δ*uvrC, rdxA::aphA3*	This study
BAC310	26695Δ*uvrD* ::*aphA3*	Moccia et al. 2012
Sbac6	J99Δ*uvrC, rdxA* ::*aphA3*	This study
SBac12	N6Δ*uvrC, rdxA* ::*aphA3*	This study
SBac4	26695*uvrC-Y18F-Y29F-D399* , *rdxA::aphA3*	This study
SBac67	26695*uvrC-ΔGIG559* , *rdxA::aphA3*	This study
SBac68	26695*uvrC-Y18F-Y29F-D399A-ΔGIG559* , *rdxA::aphA3*	This study
SBac39	26695Δ*uvrC* P*ureA:uvrC:aphA3, rdxA::Gm*	This study
SBac41	26695 P*ureA:uvrC:aphA3*	This study
