# Supplementary material for: The Helicobacter pylori UvrC Nuclease Is Essential for Chromosomal Microimports after Natural Transformation
Source: mBio. 2022 Jul 25;13(4):e01811-22. doi: 10.1128/mbio.01811-22 (PMC9426483; doi:10.1128/mbio.01811-22)
Supplement: TABLE S2 [file mbio.01811-22-s0006.pdf]

| Primer | Target gene | 5' → 3' sequence* | RS** | Application | Source |
|---|---|---|---|---|---|
| HPrdxA-1 | *rdxA* | TTTAAATTTGAGCATGGGGCAG | | Amplification of *rdxA* locus from pCJ535 and pSUS2928 for MuGENT | Bubendorfer et al. 2016 |
| HPrdxA-2 | *rdxA* | TGAAAACACCCCTAAAAGAGCG | | | Bubendorfer et al. 2016 |
| uvrC_For_PstI | *uvrC* | atactgcagAGACGCTCGTTTGCATGAAT | PstI | Amplification of *uvrC* for cloning | This study |
| uvrC_Rev_BamHI | *uvrC* | ataggatccACATCTCGCACCACCGCTT | BamHI | | This study |
| uvrC_3'mutY18F_For1 | *uvrC* | GGCGTGTTTCAATATTTTGATAAA | | Site-targeted mutagenesis of *uvrC* by inverse PCR | This study |
| uvrC_3'mutY18F_Rev1 | *uvrC* | ACTGCTGTTAGGAAGGTTTTTTA | | | This study |
| uvrC_3'mutY29F_For2 | *uvrC* | TTACTCTTTATCGGTAAGGCGA | | Site-targeted mutagenesis of *uvrC* by inverse PCR | This study |
| uvrC_3'mutY29F_Rev2 | *uvrC* | TTGGCGGTTTTTATCAAAATAT | | | This study |
| uvrC_5'mutD399A_For3 | *uvrC* | AGAAATCTTTGCCACAAGCCA | | Site-targeted mutagenesis of *uvrC* by inverse PCR | This study |
| uvrC_5'mutD399A_Rev3 | *uvrC* | ACCCTATAAGGCATGCACTCTA | | | This study |
| uvrC_HhH_For7c | *uvrC* | GAAGCCAGCGTGAAAAAATTATTGG | | Site-targeted mutagenesis of *uvrC* by inverse PCR | This study |
| uvrC_HhH_Rev7c | *uvrC* | CTTTTCTTTTAAAAGAGCGATTTGT | | | This study |
| uvrC_RT_For | *uvrC* | ATGGATTTAGCCAAACTCTA | | Check *uvrC* expression by qPCR | This study |
| uvrC_RT_Rev | *uvrC* | TCATTAGAGCAAGCGTTCAAT | | | This study |
| OE_uvrC_for_XmaI | *uvrC* | atacccgggATGAATGGCTGATTTATTGT | XmaI | Amplification of *uvrC* for overexpression and complementation | This study |
| OE_uvrC_rev_XmaI | *uvrC* | atacccgggTCATGTTTTTTCCTTTAGAT | XmaI | | This study |

*Restriction sites are in lowercase

**Restriction site
