# Supplementary material for: The Helicobacter pylori UvrC Nuclease Is Essential for Chromosomal Microimports after Natural Transformation
Source: mBio. 2022 Jul 25;13(4):e01811-22. doi: 10.1128/mbio.01811-22 (PMC9426483; doi:10.1128/mbio.01811-22)
Supplement: TABLE S3 [file mbio.01811-22-s0007.pdf]

Plasmids	Genotype
pUC19	Amp^r , Colx101, MCS within *lacZ*
pADC/aphA3	Amp^r , Km^r , pUC19 derivative containing a MCS flanked by the urease promoter and a *aphA3* cassette
pCJ535	Amp^r , Cm^r , pUC19 derivative containing *rdxA* (HP0954) disrupted by a *cat* resistance cassette
pSUS2928	Amp^r , Km^r , pCJ535 derivative containing *rdxA* (HP0954) disrupted by a *aphA3* resistance cassette
pSUSuvrA	Amp^r, pUC19 derivative containing flanking regions of *uvrA* (HP0705)
pSUS3430	Amp^r, pUC19 derivative containing flanking regions of *uvrC* (HP0821)
pSUS3424	Amp^r, pUC19 derivative containing point mutations in the nuclease domains *uvrC* (HP0821)
pSUS3435	Amp^r, pUC19 derivative containing a small deletion in the DNA-binding domain of *uvrC* (HP0821)
pSUS3436	Amp^r, pUC19 derivative containing the *uvrC* (HP0821) modifications from pSUS3424 and pSUS3435
pSUS3438	Amp^r , Cm^r , pADC/aphA3 containing *uvrC* (HP0821)
