# Supplementary material for: The Helicobacter pylori UvrC Nuclease Is Essential for Chromosomal Microimports after Natural Transformation
Source: mBio. 2022 Jul 25;13(4):e01811-22. doi: 10.1128/mbio.01811-22 (PMC9426483; doi:10.1128/mbio.01811-22)
Supplement: TABLE S4 [file mbio.01811-22-s0008.pdf]

Strain	Replicate # (independent transformation experiments)	Clone # (containing non-selected imports)	Average non-selected imports per clone
26695 wt	2	27	5.81
26695 *ΔuvrA*	1	21	5.48
26695 *ΔuvrB*	1	19	7.68
26695 *ΔuvrC*	2	24	4.04
26695 *ΔuvrD*	1	18	7.61
26695*uvrC* -Y18F-Y29F-D399A-ΔGIG559	1	35	4.63
26695*ΔuvrC* P*ureA* :*uvrC*	1	30	5.57
26695 P*ureA* :*uvrC* :aphA3	1	10	7.5
J99 wt	2	20	4.3
J99 *ΔuvrC*	1	15	2.93
N6 wt	2	27	3.39
N6 *ΔuvrC*	2	38	6.52
BCM300 wt	1	34	7.15
